# The size of cell-free mitochondrial DNA in blood is inversely correlated with tumor burden in cancer patients

**DOI:** 10.1093/pcmedi/pbz014

**Published:** 2019-10-01

**Authors:** Qin An, Youjin Hu, Qingjiao Li, Xufeng Chen, Jiaoti Huang, Matteo Pellegrini, Xianghong Jasmine Zhou, Matthew Rettig, Guoping Fan

**Affiliations:** 1 Department of Human Genetics, David Geffen School of Medicine, University of California Los Angeles, Los Angeles, CA 90095, USA; 2 Department of Pathology and Laboratory Medicine, David Geffen School of Medicine, University of California Los Angeles, Los Angeles, CA 90095, USA; 3 Department of Pathology, Duke University School of Medicine, Durham, NC 27710, USA; 4 Department of Molecular, Cell, and Developmental Biology, University of California Los Angeles, Los Angeles, CA 90095-7239, USA; 5 Department of Urology, David Geffen School of Medicine, University of California Los Angeles, Los Angeles, CA 90095, USA

**Keywords:** circulating cell-free DNA (cfDNA), tumor burden, cancer progression, liquid biopsy

## Abstract

Circulating cell-free DNAs (cfDNAs) are fragmented DNA molecules released into the blood by cells. Previous studies have suggested that mitochondria-originated cfDNA fragments (mt-cfDNAs) in cancer patients are more fragmented than those from healthy controls. However, it is still unknown where these short mt-cfDNAs originate, and whether the length of mt-cfDNAs can be correlated with tumor burden and cancer progression. In this study, we first performed whole-genome sequencing analysis (WGS) of cfDNAs from a human tumor cell line-xenotransplantation mouse model and found that mt-cfDNAs released from transplanted tumor cells were shorter than the mouse counterpart. We next analyzed blood cfDNA samples from hepatocellular carcinoma and prostate cancer patients and found that mt-cfDNA lengths were inversely related to tumor size as well as the concentration of circulating tumor DNA. Our study suggested that monitoring the size of mt-cfDNAs in cancer patients would be a useful way to estimate tumor burden and cancer progression.

## Background

Cell-free DNA fragments (cfDNAs) are DNA fragments found in human bodily fluids, such as saliva, cerebrospinal fluid, urine, and blood plasma. CfDNAs can be released by tumor cells and normal cells into the blood as a result of cell death and secretion.^1^ Those tumor-originated cfDNA molecules, known as circulating tumor DNAs (ctDNAs), contain rich information about the biological properties of cancer cells and have been used as an effective biomarker for the detection and classification of cancer.^2–4^ For example, by performing whole-genome sequencing (WGS) and whole-exome sequencing (WES) on cancer patients’ blood cfDNAs, tumor-specific point mutations and copy number variations can be identified.^5,6^ In addition, whole-genome bisulfite sequencing (WGBS) and deep WGS can measure cfDNA methylation and nucleosome positioning, respectively. These epigenetic marks on cfDNAs have been used to identify the existence and location of tumors and monitor tumor burden noninvasively by estimating the percentage of ctDNAs in the total cfDNAs.^7–9^

Besides cell nuclei, mitochondria have their own genome and can also contribute to cfDNAs. Mitochondrial cell-free DNA (mt-cfDNA) is shorter than nuclear cfDNA and is more abundant in cancer patients than healthy individuals.^10–14^ Previous studies have shown that blood mt-cfDNAs from cancer patients are shorter than those from healthy individuals, but the origination of these short mt-cfDNAs is still unknown.^10^ In addition, it is unclear whether mt-cfDNA length is predictive of other clinical symptoms.

To answer these questions, we constructed a mouse xenotransplantation model by transplanting a human prostate cancer cell line into immunodeficient mice and collected cfDNAs 2 weeks after the transplantation. By measuring mt-cfDNA length using unbiased whole-genome sequencing, we found that short mt-cfDNA fragments were released directly from the cancer cells but not from mouse tissue. Consistently in humans, our reanalysis of public datasets showed that only cancer patients and not autoimmune disease patients exhibit shorter mt-cfDNA length compared to healthy individuals. By monitoring prostate cancer patients over the course of drug treatment and re-analyze public datasets, we found that mt-cfDNA length correlates with tumor burden and cancer relapsing. Our results suggest that we may measure the size of mt-cfDNA to monitor tumor burden and cancer progression over time.

## Methods

### Animals and xenotransplantation model construction

Immunocompromised NSG (NOD.Cg-PrkdcscidIl2rgtm1Wjl/SzJ) mice from Jackson Laboratories were kept in cages under 12 h of light-dark conditions. For xenotransplantation, freshly prepared cells (2 × 10^6^) were suspended in 0.1 mL of HBSS/Matrigel (Life Technologies) mixture (1:1 V/V) and were inoculated subcutaneously (s.c.) into the flanks of 6–8-week-old mice. The use of animals was approved and guided by the Animal Research Committee of UCLA. For this study, three mice were used, and xenograft tumor occurrence was 100%. When tumor volumes reached sizes of 100–200 mm^3^ (approximately day 14 after inoculation), 0.5–1 mL of blood was collected from each mouse through cardiac puncture immediately after mouse euthanasia with carbon dioxide (CO_2_) followed by cervical dislocation.

### Collection of blood and tissue samples from metastatic castration-resistant prostate cancer patients

Six prostate cancer patients with metastatic castration-resistant prostate cancer (mCRPC) were recruited in this study. Five patients received different types of treatments, except patient No. 4 who received no treatment after being diagnosed with prostate cancer. The patients’ clinical variables are listed in Table S4. Various types of tissue samples were collected from these patients. For patients 1 through 5, we collected blood samples at multiple time points with 3-month intervals (except patient No. 4 who had only one blood sample collected). For patient No. 10, we collected blood (premortem), primary tumor tissue (postmortem), and two metastatic bone lesions (postmortem). Plasma cfDNA, genomic DNA from peripheral blood mononuclear cells, primary tumor tissue, and two bone metastatic lesions were extracted.

### Blood sample processing and cfDNA extraction

Blood samples were kept at 4 °C and processed within 30 mins after blood drawn. Briefly, the blood sample was centrifuged at 1600*g* for 10 min at 4 °C. Crude plasma was carefully transferred to a new tube without disturbing buffy coat. Crude plasma was then centrifuged again at 16 000*g* for 10 min at 4 °C, and the supernatant was carefully collected without disturbing the pellet. The resulting plasma samples were used for cfDNA extraction immediately or stored at −80 °C if not being processed immediately. CfDNA extraction was performed using QIAamp Circulating Nucleic Acid Kit (Qiagen Cat No./ID: 55114) following the manufacturer’s instructions. CfDNA were stored at −80 °C before use. Buffy coat was carefully collected using a 1 mL pipette, and genomic DNA of the buffy coat was extracted by using the standard phenol-chloroform extraction protocol.

### Genomic DNA extraction from solid tissue samples

Primary tumor tissue and two bone metastatic lesions were frozen at −80 °C until use. We first performed H&E staining on adjacent sections of tumor lesions (soft tissue such as primary tumor tissues were cryo-sectioned; bone lesions were surface-decalcified then paraffin-sectioned). The pathology specialist at UCLA helped to identify the tissue regions with a high density of tumor cells. Only samples with a high density of tumor cells were kept and benign tissues surrounding tumor lesions were carefully removed. Genomic DNA was extracted from the processed primary tumor tissue using the phenol-chloroform extraction protocol. QIAamp DNA FFPE Tissue Kit (Cat No./ID: 56404) was used to extract DNA from FFPE bone metastatic lesions.

### Sequencing library construction

A complete list of clinical samples we collected and genomic data we generated from each sample were summarized in Table S1. CfDNA WGS libraries were constructed using ThruPLEX Plasma-seq Kit (Rubicon), following the manufacturer’s instructions. Buffy coat and primary tumor genome DNA WGS libraries were constructed using the KAPA LTP library preparation kit. Specifically, genome DNA was sonicated to 350 bp using Bioruptor® Plus, and 100 ng fragmented genome DNA was inputted into KAPA LTP library preparation kit, with two cycles in the final library amplification step. The libraries were then sequenced on the Illumina HiSeq 4000 platform, in 150-bp paired-end mode.

CfDNA WGBS libraries were constructed using the Accel-NGS® Methyl-Seq DNA Library Kit (Swift Biosciences, Catalog No. 30024), following the manufacturer’s instructions. Specifically, 5 ng input cfDNA together with 0.5% w/w fragmented lambda DNA, was bisulfate converted using the EZ DNA Methylation-Direct Kit (Zymo, Catalog Nos. D5020), and converted DNA was introduced into Swift kit. Buffy coat and primary tumor genome DNA WGBS libraries were constructed using Accel-NGS® Methyl-Seq DNA Library Kit (Swift Biosciences), following the manufacturer’s instructions. Then, 50 ng fragmented genome DNA (fragmented using Bioruptor® Plus to average length 350 bp), together with 0.5% w/w lambda DNA (fragmented using Bioruptor® Plus to average length 350 bp) was introduced into the Swift kit. The libraries were then sequenced on the HiSeq 4000 platform from Illumina, using 150-bp pair-ended mode.

Whole-exome DNA was captured from total genomic DNA using the SeqCap EZ System from NimbleGen according to the manufacturer’s instructions. Briefly, genomic DNA was sheared, size selected to roughly 200–250 bp, and the ends were repaired and ligated to specific adapters and multiplexing indexes. Fragments were then incubated with SeqCap biotinylated DNA baits followed by the ligation-mediated polymerase chain reaction, and the RNA–DNA hybrids were purified using streptavidin-coated magnetic beads. The RNA baits were then digested to release the targeted DNA fragments, followed by a brief amplification of 15 or fewer PCR cycles. The libraries were then sequenced on the HiSeq 3000 platform from Illumina, using 150-bp pair-ended mode.

### Data analysis

WGS and WES sequencing data were firstly trimmed using Trim Galore!, and then mapped to human reference genome hg19 using the Burrows–Wheeler Aligner (BWA) mem algorithm. Group information was added to resulting BAM files and duplicated reads were removed using Picard. Somatic mutations were identified using GATK Mutect2 with default parameters (Buffy coat DNA in each patient was used as a normal reference when running Mutect2). Resulting somatic mutations were filtered using customized Python code, and all statistical analysis was performed using R. CfDNA fragment lengths were computed using the Picard CollectInsertSizeMetrics function.

WGBS data were firstly trimmed with Trim Galore! to remove adapter sequences as well as low-quality sequence. Resulting reads were aligned to human reference genome hg19 and lambda phage genome simultaneously using BISMARK^15^ with default parameters. CancerDetecter was run using the prostate cancer DNA methylome from TCGA with default parameters.

## Results

### Comparison of WES and WGBS in the measurement of the ctDNA percentage

Accurate determination of ctDNA percentage is of great clinical interests because it can be used to monitor cancer progression and tumor response to drug treatments. WES and WGBS are two popular methods to estimate ctDNA percentage,^2,5,8^ but no direct comparison has been made between them. To benchmark these two methods, we collected a primary tumor lesion, two bone metastatic lesions and a blood sample from patient No. 10 (a prostate cancer patient passed away at the late mCRPC stage), and generated WGBS and WES data from three tumor lesions’ genomic DNA, peripheral blood mononuclear cell (PBMC) genomic DNA, and cfDNA (Table S1). We identified 28 tumor-specific point mutations shared between solid tumor lesions and cfDNA (Table S2). We found that two bone metastases share a similar set of mutations, and cfDNA shared more mutations to metastatic lesions than the primary tumor (Fig. S1A). This similarity between metastasis and the cfDNA indicated that the metastatic lesions, instead of the primary tumor, could be the major contributor of ctDNA. To estimate the ctDNA percentage in this cfDNA sample, we determined the allele frequency of these 28 tumor-specific mutations in cfDNA and then computed their average allele frequency. Our result showed that the average allele frequency of tumor-specific mutations in this cfDNA sample was 4.80% in the haploid genome (Fig. S1B), suggesting the ctDNA percentage is 9.6% (all tumor-specific mutations we found are heterozygous). To estimate ctDNA percentage using WGBS, we generated WGBS data from the cfDNA, primary tumor, and PBMC genomic DNA, and applied CancerDetector to determine the ctDNA percentage based on DNA methylation profiles. CancerDetector used prostate cancer samples from TCGA as the tumor reference and normal plasma samples as the normal reference to deconvolute all cfDNA fragments into two groups: fragments from prostate cancer cells and fragments from normal tissues. We randomly subsampled three-quarters of reads from the normal plasma sample as the normal reference and ran the analysis 10 times. For each subsampling run, we tested different threshold in selecting cancer-specific methylation signatures, producing different sets of biomarkers. Our results showed that the ctDNA percentage in this cfDNA sample is 8.82% ± 0.28% (Table S3), a percentage that is close to the estimated value from the WES method. Taken together, our results suggest the consistency between the two prevailing methods is excellent in the estimation of ctDNA quantity.

### Short mt-cfDNAs are released by cancer cells

**Figure 1:**
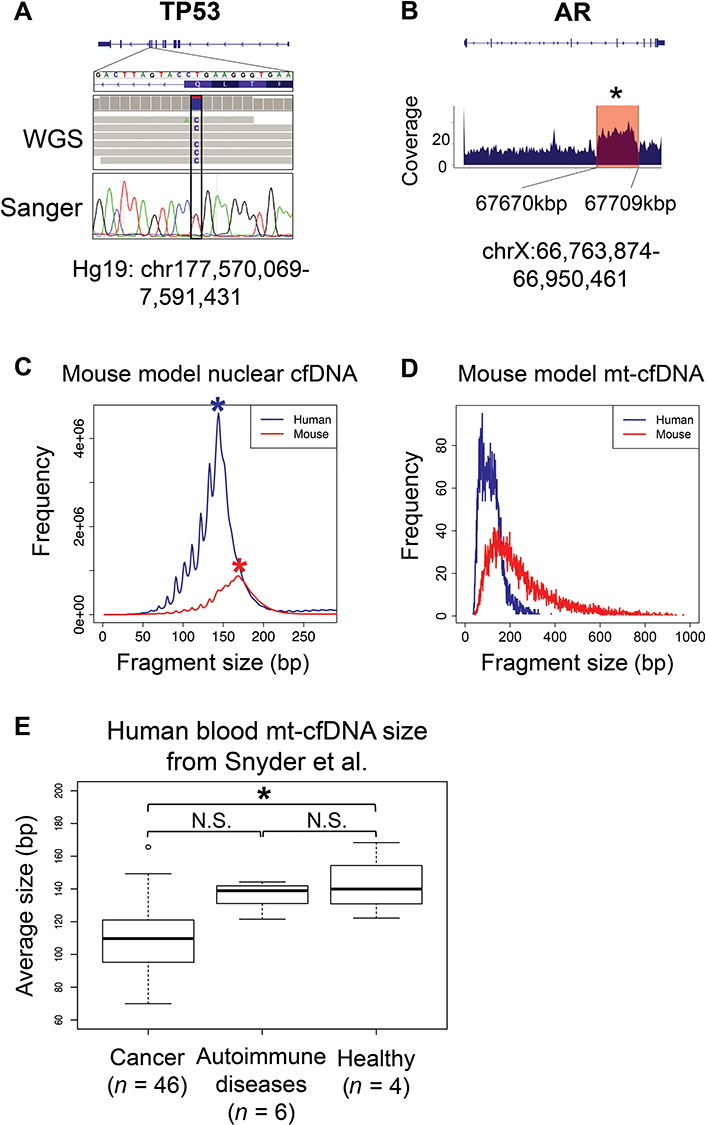
**Short mt-cfDNAs are mainly released by cancer cells. A**. From mouse xenotransplantation model blood cfDNA, we detected a point mutation on human TP53 gene, using both WGS and Sanger sequencing. This mutation is reported to be exist in the genome of CWR-R1 cancer cell line. **B**. From mouse xenograft model blood cfDNA, we detected a CNV, which is specific for CWR-R1 cancer cell line, on human AR gene. The blue shade at bottom shows the WGS reads coverage around AR genes. The red box highlights the genomic region where CNV locates. This region has significantly higher coverage compared to its flanking regions (Mann–Whitney test, *P* < 0.05). **C**. Histogram showing the size distribution of nuclear cfDNA fragments in mouse xenograft model blood cfDNA sample. Human and mouse cfDNAs were shown separately. Asteroids mark the most abundant peaks for mouse and human cfDNAs. **D**. Histogram showing the size distribution of mt-cfDNA fragments in mouse xenograft model blood cfDNA sample. The *y*-xis is the fragment number normalized by number of nuclear cfDNA fragments mapped to each genome. **E**. Boxplot showing the difference of average mt-cfDNA size between cancer patients, autoimmune disease patients and healthy human individuals. Statistic test were performed using two-tailed *t*-test (*P* < 0.05). See online supplementary material for a colour version of this figure.

Previous studies showed that blood mt-cfDNAs in cancer patients are shorter than those in healthy individuals, but it is unclear whether these short mt-cfDNA fragments are released by cancer cells or normal cells. To determine the origination of short mt-cfDNAs, we made a mouse xenotransplantation model by transplanting a well characterized human prostate cancer cell line, CWR-R1, into NOD/SCID mice. We collected blood cfDNA 2 weeks after transplantation and generated 378 million pair-end WGS on this cfDNA sample. After mapping WGS reads back to human and mouse reference genome simultaneously, we found that while only 0.015% of reads can be mapped to both the human and mouse reference genome, 287 million reads (76% of total reads) can only map to the human genome, and 72 million reads (20% of total reads) can only map to the mouse genome (Fig. S2A). The TP53 gene in CWR-R1 cell line contains a T-to-C point mutation on its 9th exon,^16^ and this mutation was detected in cfDNA using both WGS and Sanger sequencing (Fig. 1A). We also detected a 39 k-bp duplication on the AR gene as previously reported in the CWR-R1 cell line (Fig. 1B).^17^ By tiling the human reference genome into 5 kb long, non-overlapping bins, and counting reads mapped to each bin, we identified copy number variation (CNV) that were reported in the CWR-R1, such as a gain of copy number in the q arm of human chromosome 1 (Fig. S2C). Taken together, these results demonstrated that human originated cfDNA fragments in our xenotransplantation model were released by cancer cells, and these cancer cells are the main contributor of blood cfDNA in the xenotransplantation mouse model.

We then measured the fragment length of blood cfDNAs from the mouse xenotransplantation model using paired-end WGS. We first measured the cfDNA fragments originating from the nuclear genome. We found that nuclear ctDNA fragments (from the human genome) exhibited a peak at 144 bp, whereas normal cfDNA (from the mouse genome) had a peak at 169 bp. Human ctDNA size distribution presented strong 10.6 bp periodicity, and this pattern was not as evident in mouse cfDNA (Fig. 1C). These results were highly consistent with what had been reported before.^18,19^ Our unbiased WGS captured 32 549 mt-cfDNAs from the human genome and 16 785 mt-cfDNAs from the mouse genome. We then sought to compare the mt-cfDNA size released by cancer cells or normal tissue cells. By measuring the mt-cfDNA fragment size, we found significantly shorter mt-cfDNA fragments released from human cancer cells than those from normal mouse tissues (Fig. 1D). Taken together, these results suggested that cancer cells, but not normal tissue, are the main origination of shorter mt-cfDNA fragments.

To further examine the mt-cfDNA size in human patients, we reanalyzed the dataset generated by Snyder *et al.*,^7^ and accessed the mt-cfDNA sizes between cancer patients, autoimmune disease patients, and healthy individuals. Consistently, we found that the average length of mt-cfDNAs from cancer patients (average fragment length: 109.15 bp, *n* = 46) were significantly shorter than mt-cfDNAs from healthy controls (average fragment length: 142.62 bp, *n* = 4) (Fig. 1E) (*t*-test, *P*-value < 0.05). Interestingly, although both autoimmune disease patients and cancer patients have elevated cfDNA abundance in their blood, autoimmune disease patients have similar mt-cfDNA size compared to healthy individuals (Fig. 1E). These discoveries further support the conclusion that cancer cells, but not normal tissue, are the main origination of shorter mt-cfDNA fragments.

### Mt-cfDNA length is inversely correlated with tumor size and circulating tumor DNA concentration in hepatocellular carcinoma patients

**Figure 2:**
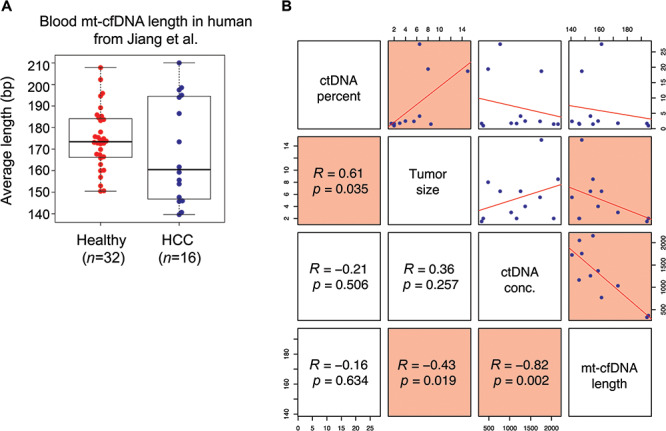
**Mt-cfDNA length correlates with tumor size and circulating tumor DNA concentration. A.** Beeswarm-boxplot showing the average mt-cfDNA size between healthy individuals and hepatocellular carcinoma patients. **B**. Scatterplot matrix representing the Pearson correlation between average mt-cfDNA length and (1) percentage of circulating tumor DNA out of total cfDNA, (2) tumor size and (3) circulating tumor DNA concentration in blood. Significant correlations are highlighted in red. The ctDNA percentage of 12 samples were derived from Figure 3 of the paper by Jiang *et al.*^11^ See online supplementary material for a colour version of this figure.

As we have observed that mt-cfDNA size is significantly shorter in cancer patients compared to healthy individuals or patients with autoimmune diseases, we asked whether mt-cfDNA size correlates with other clinical parameters related to cancer. By reanalyzing the WGS data of cfDNA samples collected from hepatocellular carcinoma patients by Jiang *et al.*,^11^ we observed that mt-cfDNA size appears to be shorter in cancer patients compared to that in healthy individuals, but no statistical significance were found with this dataset, due to high variance in HCC samples (Fig. 2A). Specifically, the blood mt-cfDNA fragments from hepatocellular carcinoma patients (average fragment length: 162.41 bp, *n* = 16) are shorter than those of healthy controls (average fragment length: 173.25 bp, *n* = 32). In addition, patients with high ctDNA abundance (ctDNA percentage > 5%) had shorter mt-cfDNA fragments (average fragment length: 153.62 bp) compared to patients with low ctDNA abundance (ctDNA percentage < 5%, average fragment length: 164.36 bp) (*t*-test, *P* < 0.05). The percentage of ctDNA out of total cfDNA significantly correlates with tumor size (Fig. 2B). Importantly, we found that mt-cfDNA size is inversely correlated with tumor size and ctDNA concentration in the blood (Fig. 2B) (Pearson correlation test, *P* < 0.05). Taken together, these reanalysis results suggest that the size of mt-cfDNA is a useful parameter that correlates with tumor burden and ctDNA concentration.

### Mt-cfDNA length is inversely correlated to the degree of metastatic castrate-resistant prostate cancer progression

**Figure 3:**
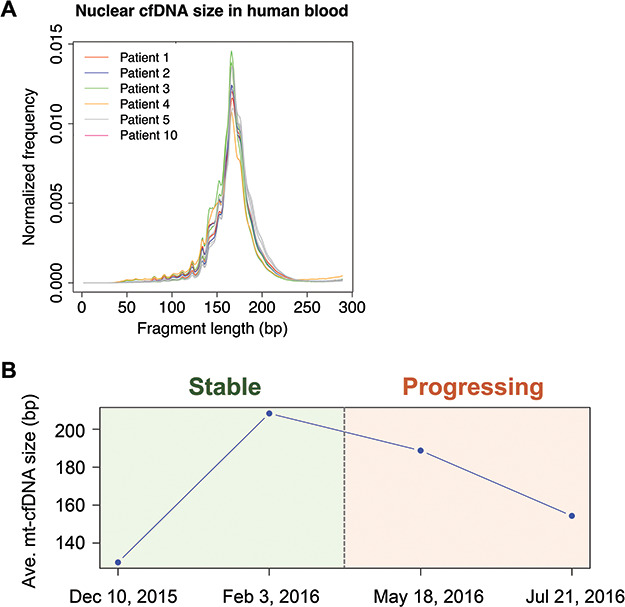
**Mt-cfDNA length correlates with metastatic castrate-resistant prostate cancer progression. A.** Histogram showing the size distribution of nuclear cfDNA fragments in 12 blood samples from six mCRPC patients. **B.** The line plot shows the average mt-cfDNA size across four time points (with 3-month interval in between) in mCRPC patient No. 5. The background color marks the disease state for each timepoint. See online supplementary material for a colour version of this figure.

We collected 12 blood samples from six metastatic castrate-resistant prostate cancer (mCRPC) patients across multiple time points together with a primary tumor lesion and two bone metastatic lesions, and generated WGS, WGBS, and WES data from them (Table S1 and Table S4). By examining the nuclear cfDNA size using WGS data, we found that cfDNA samples from different prostate cancer patients have very similar length distribution, with global-maximal peaks at 165–167 bp (Fig. 3A). We also observed a series of local-maximum peaks from 50 bp to 150 bp in length, with 10.6 bp interval between each pair of adjunct peaks. Overall, these results were highly consistent with the results previously reported in human cfDNA samples, confirming the reliability of our WGS data for measuring cfDNA size.^18,20^

We then measured mt-cfDNA length in these cfDNA samples. Notably, we tracked one mCRPC patient (patient No. 5) for 8 months over four time points. The disease state was stable at the first two time points but began progressing after the second time point. Interestingly, we found that the mt-cfDNA size initially increased, but then decreased after the second time point (Fig. 3B). Taken together, these results suggest that mt-cfDNA size in blood can be correlated with mCRPC progression status.

## Discussion

Recently, several studies have investigated the blood mt-cfDNA size in cancer patients using WGS or qPCR-based methods and showed that mt-cfDNAs are more fragmented in cancer patients compared to healthy individuals.^13,21^ However, the origination of short mt-cfDNA in cancer patients is still unclear. In this study, using WGS data of blood cfDNA from a mouse xenotransplantation model and human individuals, we showed that cancer cells are the source of short mt-cfDNA fragments. Furthermore, mt-cfDNA size is predictive for tumor size and ctDNA concentration in blood in hepatocellular carcinoma patients. Compared to the prevailing methods for measuring tumor burden such as diagnostic imaging, liquid biopsy for cancer screening is less invasive and potentially cost effective. By monitoring cfDNAs at different time points from an mCRPC patient, we found mt-cfDNA could be correlated with cancer progression, and shorter mt-cfDNA is associated with a bigger cancer burden. Thus, the size of mt-cfDNA could be a biomarker for the prognosis for cancer progression.

Although we have evidence that short mt-cfDNA comes from cancer cells, it is unclear why cancer cells can release short mt-cfDNA fragments. It is known that cfDNA is passively released from cells undergoing apoptosis and necrosis.^21^ Previous studies using different cell lines showed that the mitochondrial genome DNA is actively degraded when cells undergo necrosis.^22^ On the contrary, mitochondrial DNA remains intact after apoptosis. In addition, the mitochondrial membrane remains morphologically intact after apoptosis, whereas it was ruptured during necrosis.^23^ DNA inside intact mitochondria could be protected from enzymes in blood such as RNA inside exosomes, and remains relatively intact. Therefore, the relative short mt-cfDNAs we observed are more likely to be released by cells undergoing necrosis than apoptosis. Since we found that short mt-cfDNAs are mainly released by tumor cells, this raises the possibility that tumor cells *in vivo* are likely to undergo necrosis compared to normal cells, thus releasing more fragmented mitochondrial DNA into blood. In addition, necrosis inside tumor increases when tumor size enlarges,^24^ and this observation is consistent with our result that mt-cfDNA size inversely correlates with the tumor size.

Previous studies suggest that ctDNA percentage out of total cfDNA can reflect tumor burden and disease progression, and both WES- and WGBS-based methods have been used to estimate ctDNA percentage.^6,8,25,26^ We evaluate the consistency of these two methods by collecting blood cfDNA, PBMC genomic DNA, primary tumor DNA, and genomic DNA from two metastatic lesions from one mCRPC patient for high coverage WGS, WGBS, and WES assays (Table S1). Although both WES and WGBS gave a reasonable estimate of ctDNA percentage for this patient, the percentages estimated by WES (9.6%) or by WGBS (8.9%) are slightly different from each other. Nevertheless, our results suggest that either WES or WGBS would provide a good estimate of ctDNA quantity in totaldraftrules cfDNA.

This current study has certain limitations that are worth pointing out. First, due to the size preference during WGS library construction and sequencing, very short (< 100 bp) and very long (> 1000 bp) cfDNA fragments are underrepresented in our sequencing data. Optimized sequencing methods or quantitative-PCR based methods can be used to address this technical hurdle.^19^ Second, although our result from mouse xenotransplantation model showed that short mt-cfDNA are mostly released by human cancer cells and suggested short mt-cfDNA could be mainly released by tumor cells, it should be noted that human and mouse cells potentially contain species-specific nucleases that may lead to shortening of mt-cfDNA in human cells. Finally, we realize that our results are limited by relatively a low number of patient samples. Future study with a larger number of patient samples is warranted to further validate our conclusions.

While this paper was being peer-reviewed, Cristiano *et al.* reported the blood cfDNA fragmentation pattern can be noisy in cancer patients compared to healthy individuals.^27^ In fact, we also found the nuclear cfDNA fragmentation pattern of patient 4, a mCRPC patient received no treatment after being diagnosed, has a much nosier fragmentation pattern when compared to other mCRPC patients (Fig. S3A). Consistently, in patient 5, the patient we tracked for four time points over 8 months, we found the nuclear cfDNA fragmentation patterns are flat at the first two time points, but become much nosier after the second time point (Fig. S3B). The disease state in patient 5 was stable at the first two time points but began progressing after the second time point. Although Cristiano *et al.* did not report any correlation between nuclear cfDNA fragmentation pattern and cancer progression, our results from the patient 5 case suggest the potential utility of cfDNA fragmentation pattern in monitoring cancer progression.

## Supplementary Material

Supplementary_figures_for_pbz014

Tables_V7_R2_pbz014

## Data Availability

All WGBS, WGS and WES data generated in this study are available upon reasonable request to the author.
