# Supplementary material for: The size of cell-free mitochondrial DNA in blood is inversely correlated with tumor burden in cancer patients
Source: Precis Clin Med. 2019 Oct 1;2(3):131–9. doi: 10.1093/pcmedi/pbz014 (PMC6770274; doi:10.1093/pcmedi/pbz014)
Supplement: Supplementary_figures_for_pbz014 [file supplementary_figures_for_pbz014.docx]

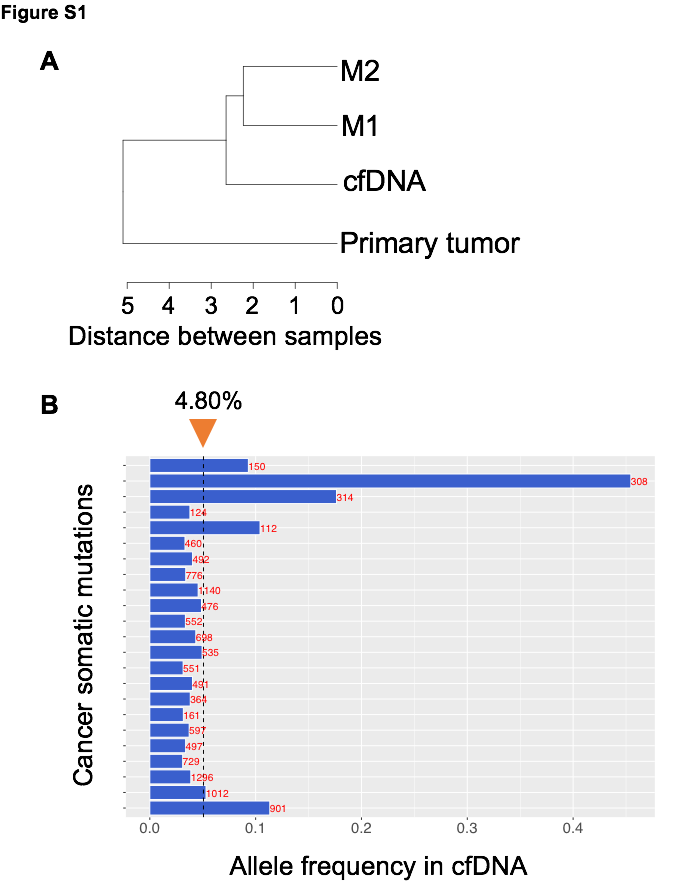


**Figure S1.** Analysis results from WES data generated from samples collected from patient 10**. A.** Hieratical clustering tree representing similarity of sample based on their mutation landscape. Two bone metastasis are similar to each other and cfDNA are closer to bone metastasis than primary tumor. **B.** Allele frequency of tumor-specific point mutations in cfDNA estimated by WES data. High confident point mutations were identified form 3 solid tumor lesions WES dataset and their allele frequency is shown as height of the bars. The red number on the top of each bar represents how many reads covers this mutation in cfDNA. ctDNA percentage is computed by 2 times the average allele frequency of all sites. Average allele frequency of tumor-specific mutations in this cfDNA sample was 4.80%, suggesting that the ctDNA percentage is 9.6%.


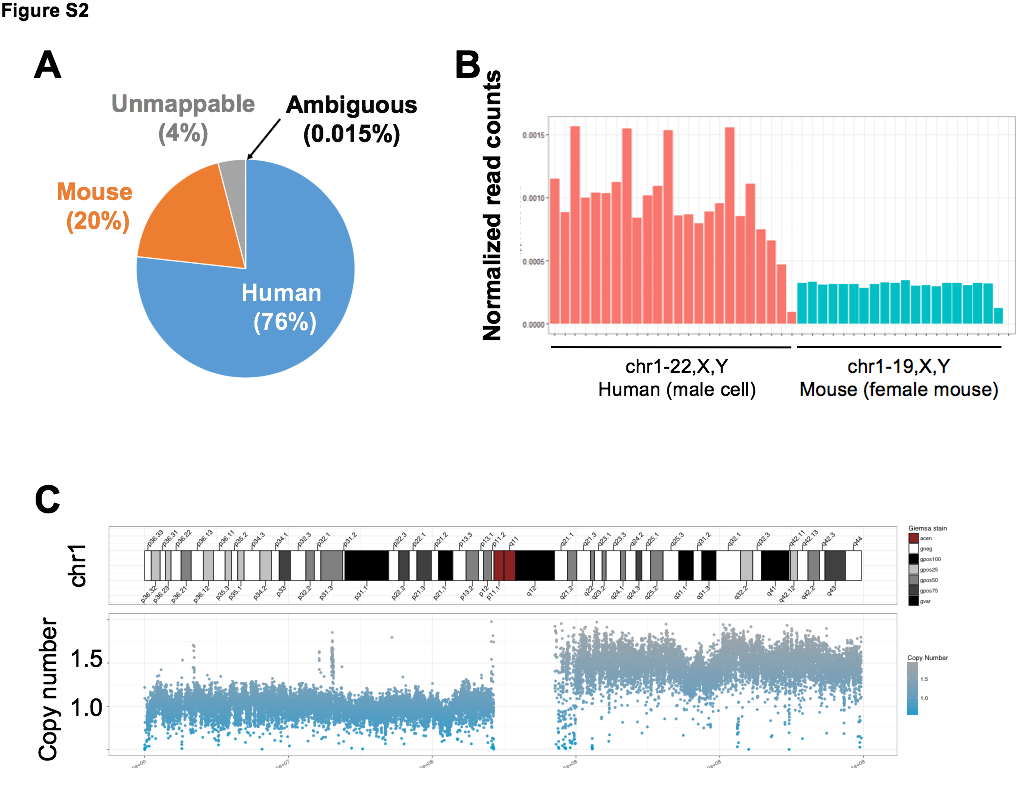


**Figure S2.** Analysis of mouse xenograft model cfDNA WGS data. **A.** Pie chart showing the percentage of reads mapped to mouse genome only (20%), mapped to human genome only (76%), mapped to both genome (0.015%) and cannot be mapped anywhere (4%). **B**. Bar plot showing the normalized read number mapped to each genome. The height of each bar shows the number of reads mapped to each chromosome divided by the length of the chromosome. **C.** Gain of one copy number on human chromosome 1 q arm, which has been reported to exist in CWR-R1 cell line. The dot plot at the bottom shows the read count for 5 kbp bins on chromosome 1. Each dot represents one bin. The copy number is inferred using the mean coverage among all human chromosomes.


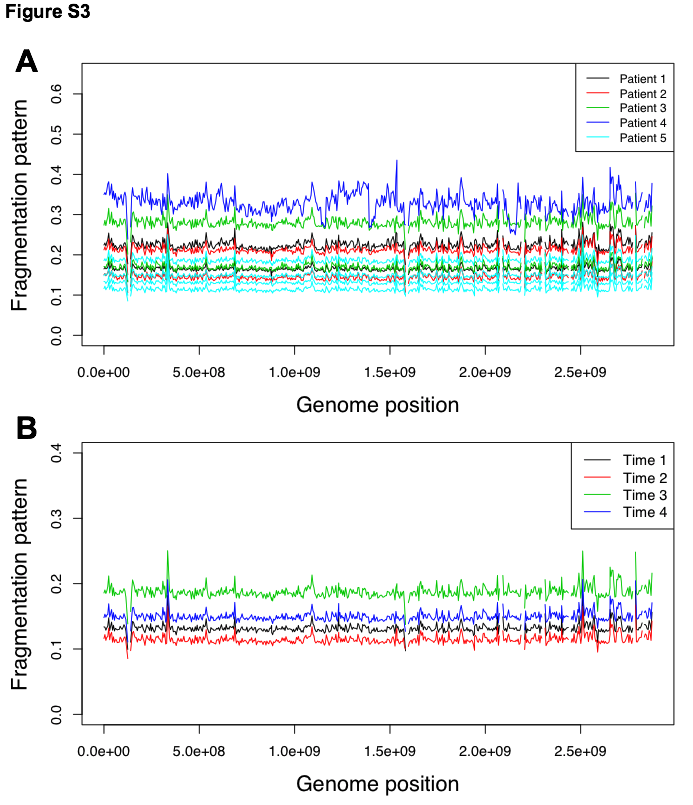


**Figure S3.** Aberrant and noisy genome-wide fragmentation pattern of blood cfDNA in mCRPC patients. **A.** Genome-wide blood cfDNA fragmentation pattern was computed for all mCRPC patient samples, following the method described by Cristiano et al. ^1^ Briefly, autosomes were tiled into 5 Mb long bins, excluding centromeres and mappability blacklist regions. Bins with lowest 10% number of reads mapped were removed. Fragmentation pattern was defined as the ratio of short (100-150 bp long) to long fragments (longer than 150 bp). Using this method, we found that patient 4, a mCRPC patient receiving no treatment after being diagnosed with protonate cancer, has the nosiest fragmentation pattern compared to all other mCRPC patients treated with different therapy. **B**. Fragmentation pattern in patient 5 across 4 time points. The fragmentation pattern is flat at the first two time point. But starting from time point 3 when the cancer becomes progressive, the pattern becomes much nosier.

**Reference**

1. Cristiano S, Leal A, Phallen J, *et al*. Genome-wide cell-free DNA fragmentation in patients with cancer. *Nature* 2019; **570**:385–9. doi: [10.1038/s41586-019-1272-6](https://doi.org/10.1038/s41586-019-1272-6).
